# Network pharmacology-based analysis of Resinacein S against non-alcoholic fatty liver disease by modulating lipid metabolism

**DOI:** 10.3389/fnut.2023.1076569

**Published:** 2023-02-14

**Authors:** Fei-Fei Mao, Shan-Shan Gao, Yan-Jie Huang, Nian Zhou, Jin-Kai Feng, Zong-Han Liu, Yu-Qing Zhang, Lu-Yun Yuan, Gang Wei, Shu-Qun Cheng

**Affiliations:** ^1^Tongji University Cancer Center, Shanghai Tenth People’s Hospital, School of Medicine, Tongji University, Shanghai, China; ^2^Guangdong Cardiovascular Institute, Guangdong Provincial People’s Hospital, Guangdong Academy of Medical Sciences, Guangzhou, Guangdong, China; ^3^Department of Hepatic Surgery VI, Eastern Hepatobiliary Surgery Hospital, Second Military Medical University, Shanghai, China; ^4^Cancer Center, Yue Yang Hospital of Integrative Traditional Chinese and Western Medicine, Affiliated to Shanghai University of Traditional Chinese Medicine, Shanghai, China; ^5^Beijing Key Laboratory of Diabetes Research and Care, Department of Endocrinology, Beijing Diabetes Institute, Beijing Tongren Hospital, Capital Medical University, Beijing, China

**Keywords:** *Ganoderma resinaceum*, Resinacein S, NAFLD, lipid metabolism, network pharmacology

## Abstract

**Background:**

*Ganoderma lucidum* is reportedly the best source of traditional natural bioactive constituents. Ganoderma triterpenoids (GTs) have been verified as an alternative adjuvant for treating leukemia, cancer, hepatitis and diabetes. One of the major triterpenoids, Resinacein S, has been found to regulate lipid metabolism and mitochondrial biogenesis. Nonalcoholic fatty liver disease (NAFLD) is a common chronic liver disease that has become a major public health problem. Given the regulatory effects on lipid metabolism of Resinacein S, we sought to explore potential protective effects against NAFLD.

**Methods:**

Resinacein S was extracted and isolated from G. *lucidum*. And mice were fed with high fat diet with or without Resinacein S to detect hepatic steatosis. According to Network Pharmacology and RNA-seq, we analyzed the hub genes of Resinacein S against NAFLD disease.

**Results:**

Our results can be summarized as follows: (1) The structure of Resinacein S was elucidated using NMR and MS methods. (2) Resinacein S treatment could significantly attenuate high-fat diet (HFD)-induced hepatic steatosis and hepatic lipid accumulation in mouse. (3) GO terms, KEGG pathways and the PPI network of Resinacein S induced Differentially Expressed Genes (DEGs) demonstrated the key target genes of Resinacein S against NAFLD. (4) The hub proteins in PPI network analysis could be used for NAFLD diagnosis and treatment as drug targets.

**Conclusion:**

Resinacein S can significantly change the lipid metabolism in liver cells and yield a protective effect against steatosis and liver injury. Intersected proteins between NAFLD related genes and Resinacein S-induced DEGs, especially the hub protein in PPI network analysis, can be used to characterize targets of Resinacein S against NAFLD.

## 1. Introduction

*Ganoderma lucidum* is a medicinal mushroom that can prolong life and promote health and has a long history in traditional Chinese medicine. Given its growing consumption, it has been intensively planted and sold since the 1970s. It is widely thought to be effective in preventing and treating many diseases and has anti-cancer properties (1). *G. lucidum* is considered the best source of traditional natural bioactive components. It contains various compounds, including polyphenols, polysaccharides, steroids, triterpenes, nucleotides, amino acids, trace elements, and vitamins. Over the past few years, *G. lucidum* extract has been used as a dietary addition to treating various diseases (2). Among various active components of *G. lucidum*, polysaccharides (GL-PS) and terpenoids (GL-T) predominantly exert physiological activities. They can inhibit the cell cycle and yield cytotoxicity and anti-metastasis, immunomodulation, antioxidant, antibacterial, anti-inflammatory and other effects (3). Moreover, *G. lucidum* has been recognized as an alternative adjuvant for treating leukemia, cancer, hepatitis and diabetes (4). Current evidence suggests that *Ganoderma* triterpenes represent one of the main active ingredients of mushrooms and yield an inhibitory effect on adipogenesis, leading to decreased lipid synthesis and accumulation. Other studies revealed that *G. lucidum* extracts and ethanol extracts of chigger mites rich in triterpenes contribute to adipogenesis and adipocyte differentiation. *Ganoderma resinaceum* is generally utilized for treating hepatitis, hyperglycemia, and dysimmunity in China and Nigeria (5).

Previous researchers had separated four new triterpenoids and four identified triterpenoids with anti-obesity effects from *G. resinaceum*. What’s more, one of the triterpenoids, Resinacein S has been found to induce beige and brown phenotypes, which may be relevant to the activation of AMPK/PGC1α signaling pathway to inhibit and treat obesity and relevant diseases. At the molecular level, Resinacein S treatment could significantly induce the expression of genes and/or proteins related to thermogenesis, fatty acid oxidation and lipolysis (6). Resinacein S, as one of the major triterpenoids from *G. resinaceum*, provides a therapeutic strategy for lipid metabolic diseases such as NAFLD, but whether Resinacein S treatment could provide protective aspects against NAFLD remains unknown.

Nonalcoholic fatty liver disease (NAFLD) is currently recognized as the most common liver disease in the world, affecting about 25% of adults worldwide. It encompasses steatosis simplex to nonalcoholic steatohepatitis, fibrosis, cirrhosis, and hepatocellular carcinoma. The clinical manifestation of nonalcoholic steatohepatitis (NASH) is a serious form of nonalcoholic fatty liver disease (NAFLD) characterized by the accumulation (steatosis) of triglycerides in liver cells, inflammation, injury and apoptosis, which may lead to cirrhosis and liver cancer in extreme cases (7–9). According to the regional epidemiological model of NAFLD, economy, environment and lifestyle are the key factors of disease progression (10). Hepatic steatosis or fatty liver refers to the increase of lipid accumulation in liver cells caused by increased production or decreased clearance of hepatic triglycerides or fatty acids (11). It has been established that Resinacein S can reduce fat and triglyceride accumulation by inducing the expression of genes and/or proteins related to thermogenesis, fatty acid oxidation and lipolysis at the molecular level. Nevertheless, the protective aspects and molecular mechanisms underlying the ability of Resinacein S to target NAFLD development remain to be illustrated. Therefore, this study aimed to explore the protective effects of Resinacein S against NAFLD.

## 2. Materials and methods

### 2.1. General experimental procedures

The NMR spectra were recorded on Bruker AV500 and AVIII600 instruments, with TMS used as an internal benchmark. Optical rotations were measured on an Autopol VI-91058 digital polarimeter. The UV spectra were detected by a UV-2700 spectrophotometer, while IR spectra were measured on a Nicolet iS10 spectrometer. HR-TOF ESI/MS was conducted on an Agilent 6,200 Q-TOF MS system spectrometer. Column chromatography was carried out with silica gel, reversed-phase C18 silica gel and Sephadex LH-20 as packing materials. Fractions were quantitated by thin-layer chromatography after spraying with sulphuric acid and heating. TLC was carried out on silica gel GF254. HPLC was performed on an Agilent 1,100 liquid chromatography system coupled with a diode-array detector and an Agilent Zorbax SB-C18 column (5 μm, 9.4 × 250 mm).

### 2.2. Fungal material

Fruiting bodies of *G. resinaceum* were collected from Vientiane, Laos, identified by Peigui Liu and a voucher specimen (accession number: KGR-201606) was deposited at the State Key Laboratory of Phytochemistry and Plant Resources in West China, Kunming Institute of Botany, Chinese Academy of Sciences, China (6).

Extraction and isolation procedures have been described before (6).

### 2.3. Animal treatments and histological evaluation

Four-week-old male C57BL/6 mice were kept under the standard environment. Mice were fed with high fat diet with or without Resinacein S (intraperitoneal injection, interval for 48 h) at a dose of 10 mg/kg/day for 15 weeks (9). Then mice were sacrificed and the liver were fixed in 4% formaldehyde for HE staining and Oil Red O (G1262, Solarbio, China) staining (abs7049, Absin Bioscience, China). Images were acquired with a light microscope (Zeiss, Germany). Triglycerides (TG) were detected by a commercial kit (E1025-105, Applygen, Beijing, China) according to manufacturer’s instructions. All the experiments were approved by the ethics committee of Shanghai tenth People’s Hospital, Shanghai, China.

### 2.4. Collection and sorting of nonalcoholic fatty liver disease disease-related genes

Genes related to NAFLD were acquired from DisGeNET,^1^ GeneCards,^2^ and OMIM^3^ databases. The keyword “nonalcoholic fatty liver disease” was entered into the above three databases to search for genes related to NAFLD. Then, the intersection was obtained using the Venny Venn diagram tool (version 2.1.0).^4^

### 2.5. Determination of relevant target of Resinacein S

First, the canonical SMILES structure of Resinacein S was obtained through PubChem.^5^ Later, canonical SMILES information of Resinacein S was input into the SwissTargetPrediction^6^ database. Finally, the Uniprot ID obtained from the TargetNet database was transformed into Gene ID in UniProt^7^ database, yielding a total of 124 target genes associated with Resinacein S.

### 2.6. Protein–protein interactions network construction

The above Resinacein S targets of NAFLD were imported in String,^8^ with the organization parameter and threshold of combination score being set as Homo sapiens and 0.7 for obtaining protein–protein interactions (PPI). Then, the protein interaction network data from the STRING database was incorporated into Cytoscape 3.9.0 for classification and mapping according to the degree value.

### 2.7. RNA-seq analysis

TRIzol reagent was utilized for extracting total cellular RNA, which was later subject to spectrophotometry and agarose gel electrophoresis (AGE) with the NanoDrop ND-1000 instrument to analyze RNA integrity. Using a KAPA Stranded RNA-seq Library Prep Kit, this study built an RNA library, while a 2100 Bioanalyzer was employed for library quality assessment. Quantification of the library was performed by qRT-PCR. NCBI Gene Expression Omnibus was utilized to import raw RNA-seq information.

### 2.8. Functional analysis of identified genes

FastQC v0.11.8 was applied to analyze the raw RNA-seq data. Fragments per kilobase of gene/transcript model per million mapped fragments (FPKM) values of diverse genes and transcripts were determined by the Ballgown package of R v2.10.0 software. Additionally, R was used to generate volcano plots and heatmaps to further analyze the gene expression profiles.

Gene Ontology (GO) enrichment of Differentially Expressed Genes (DEGs) was conducted to identify significantly enriched GO-biological processes (BP), GO-molecular functions (MF) and GO-cellular components (CC). Meanwhile, DEG-related pathways were identified based on the Kyoto Encyclopedia of Genes and Genomes (KEGG) database (12).

### 2.9. Statistical analysis

All data were expressed as mean ± standard deviation (SD). Experiments were repeated at least three times. The statistical significance of differences was evaluated using either the Student’s unpaired *t*-test, One-way analysis of variance (ANOVA), or two-way ANOVA as indicated. A *p*-value <0.05 was statistically significant.

## 3. Results

### 3.1. Structure elucidation of Resinacein S

**Figure 1:**
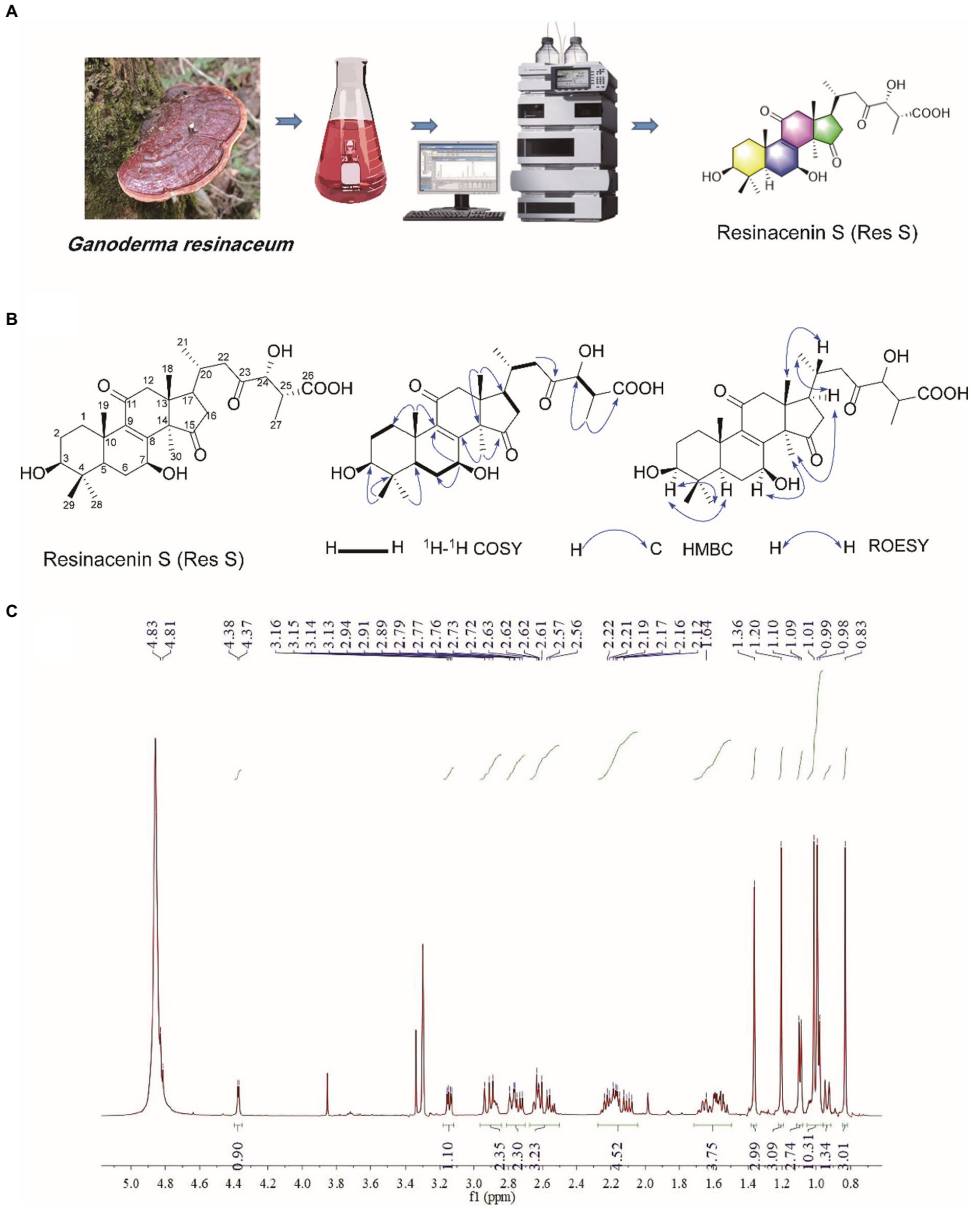
Resinacein S was obtained from *Ganoderma resinaceum* **(A)**, and ^1^H–^1^H COSY correlations, key HMBC, ROESY correlations for Resinacein S **(B)**, and ^1^H NMR spectrum (600 MHz) of Resinacein S in CD_3_OD **(C)**.

Resinacein S is a natural compound that has only been documented in *G. resinaceum*. We obtained Resinacein S white powder from the ethanol extracts of *G. resinaceum* through a series partition and column chromatography as previously described (Figure 1A) (13). Resinacein S was established as C_30_H_44_O_8_ based on the HRESIMS (Supplementary Figure S6), and the X-ray crystallographic structure of Resinacein S was obtained (Figure 1B). The ^1^H NMR and ^13^C NMR data of Resinacein S are shown in Table 1. The ^1^H NMR, ^13^C NMR and DEPT, HSQC, HMBC, ^1^H-^1^H COSY, and ROESY spectra are displayed in Figures 1B,C and Supplementary Figures S1–S6. Resinacein S was elucidated as (6R)-6-((3S,5R,7S,10S,13R,14R,17R)-3,7-dihydroxy-4,4,10,13,14-pentamethyl-11,15-dioxo-,3,4,5,6,7,10,11,12,13,14,15,16,17-tetradecahydro-1H-cyclopenta[*a*]phenanthren-17-yl)-3-hydroxy-2-methyl-4-oxo heptanoic acid.

**Table 1:** ^1^H NMR and ^13^C NMR Data (*δ*) for Resinacein S (600 MHz) in CD_3_OD (*δ* in ppm, *J* in Hz).

Position	Resinacein S		Position	Resinacein S	
	*δ*_C_, type	*δ*_H_, (*J* in Hz)		*δ*_C_, type	*δ*_H_, (*J* in Hz)
1	35.9, CH_2_	2.78, overlapped; 1.01, overlapped	16	41.8, CH_2_	2.10, dd (19.3, 9.3); 2.74, dd (19.3, 7.8)
2	28.2, CH_2_	1.65, m; 1.56, overlapped	17	46.8, CH	2.17, dd (18.5, 9.3)
3	78.9, CH	3.14, dd (11.7, 4.5)	18	17.8, CH_3_	0.99, s
4	39.7, C		19	18.8, CH_3_	1.20, s
5	50.3, CH	0.96, d (13.0)	20	32.6, CH	2.23, overlapped
6	28.0, CH_2_	1.56, overlapped; 2.17, overlapped	21	20.2, CH_3_	0.98, d (6.8)
7	67.9, CH	4.83, overlapped	22	46.4, CH_2_	2.55, dd (17.7, 8.5); 2.62, overlapped
8	158.9, C		23	212.5, C	
9	144.1, C		24	78.7, CH	4.37, d (5.0)
10	39.9, C		25	43.3, CH	2.87, overlapped
11	200.4, C		26	177.7, C	
12	51.5, CH_2_	2.92, d (16.7); 2.62, d (16.7)	27	11.6, CH_3_	1.09, d (7.0)
13	46.7, C		28	28.7, CH_3_	1.01, s
14	60.4, C		29	16.2, CH_3_	0.83, s
15	218.3, C		30	24.9, CH_3_	1.36, s

### 3.2. Resinacein S alleviated liver damage and hepatic lipid accumulation

**Figure 2:**
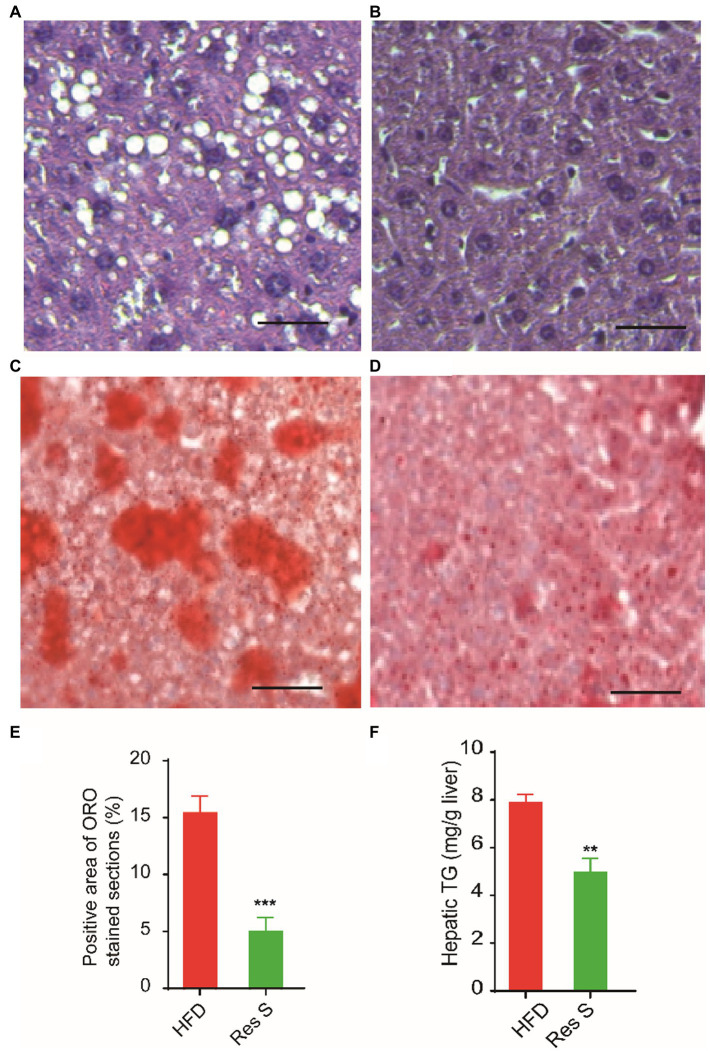
Resinacein S reduced liver damage and hepatic steatosis in HFD-fed mice. **(A,B)** H&E staining of livers in HFD-fed mice **(A)** and Resinacein S treated HFD-fed mice **(B)**. **(C–E)** Oil Red O staining of livers in HFD-fed mice **(C)** and Resinacein S treated HFD-fed mice **(D)**. **(E)** is the quantitative analysis of Oil Red O staining. **(F)** TG levels in livers of Resinacein S treated or not HFD-fed mice were detected. Values were means ± SD, and for statistical analysis, one-way ANOVA were performed between indicated groups.

To investigate the benefits of Resinacein S against the HFD-induced extraphysiological process of NAFLD, we employed Resinacein S to treat HFD-diet mice and found that Resinacein S could effectively reduce liver damage and hepatic steatosis in HFD-fed mice (Figures 2A,B). In addition, Oil Red O staining of livers in Resinacein S-treated HFD-fed mice yielded few lipid droplets of small size. Compared to the control, the livers of Resinacein S-treated mice displayed an almost normal phenotype (Figures 2C–E). Consistently, significantly lower TG levels were found in the livers of the Resinacein S-treated group (Figure 2F).

### 3.3. Target recognition results of Resinacein S and nonalcoholic fatty liver disease

**Figure 3:**
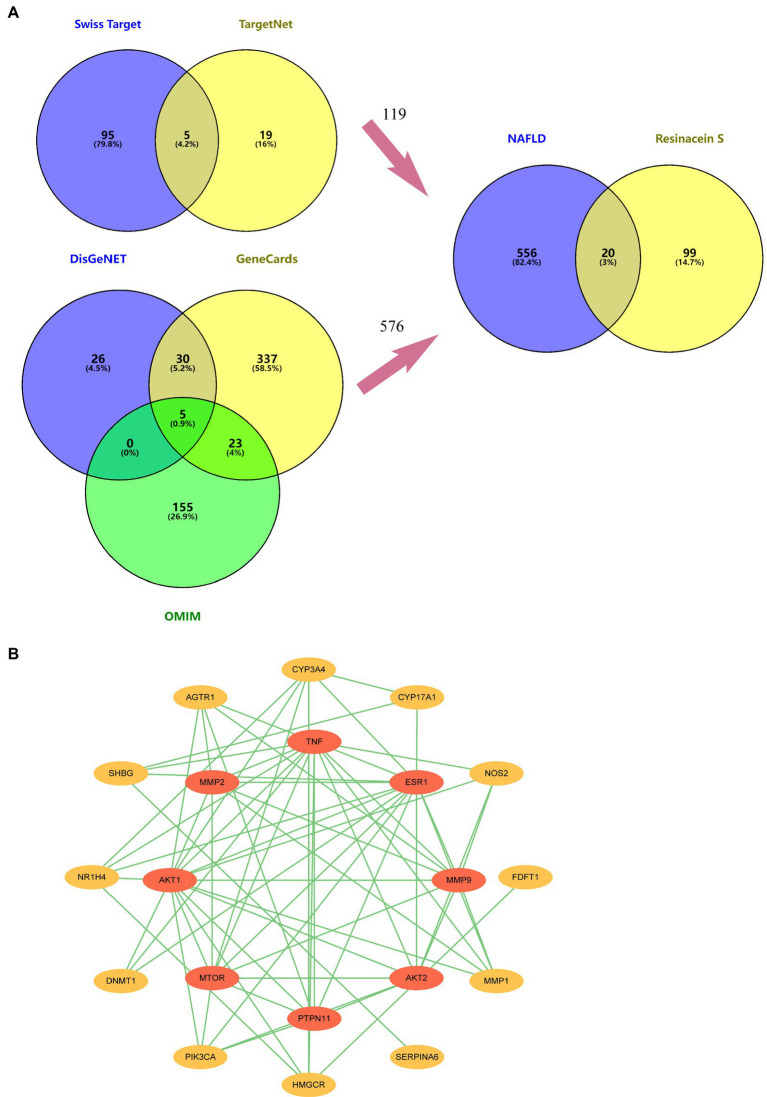
**(A)** 119 drug treatment targets based on the examination and combination of Swiss Target Prediction and TargetNet databases. Five hundred and seventy six genes related to NAFLD based on the examination and combination of DisGeNET, GeneCards and OMIM databases. There are 20 intersection targets between NAFLD and Resinacein S. **(B)** PPI protein interaction network diagram, in which the innermost circle degree value is 0–6, and the outer circle degree value is 7–16.

Twenty and 101 gene targets in human related to Resinacein S were obtained from SwissTargetPrediction and TargetNet databases, respectively. Moreover, 61, 395 and 183 gene targets related to NAFLD disease were obtained from DisGeNET, GeneCards and OMIM, respectively. The Venny 2.1.0 online system was used for target recognition (Figure 3A). One hundred nineteen action targets of Resinacein S were acquired, along with 576 NAFLD related genes. Finally, 20 action targets of Resinacein S against NAFLD come to light. Detailed information on the action targets is provided in Table 2.

**Table 2:** Twenty key target genes of Resinacein S related to NAFLD.

Genes	Protein names	UniProt ID
NR1H4	Bile acid receptor	Q96RI1
CYP17A1	Cytochrome P450 17A1	P05093
MMP1	Matrix metalloproteinase 1	P03956
FDFT1	Squalene synthetase	P37268
TNF	TNF-alpha	P01375
AGTR1	Type-1 angiotensin II receptor (by homology)	P30556
AKT1	Serine/threonine-protein kinase AKT	P31749
AKT2	Serine/threonine-protein kinase AKT2	P31751
CYP3A4	Cytochrome P450 3A4	P08684
DNMT1	DNA (cytosine-5)-methyltransferase 1	P26358
ESR1	Estrogen receptor alpha	P03372
HMGCR	HMG-CoA reductase	P04035
MMP2	Matrix metalloproteinase 2	P08253
MMP9	Matrix metalloproteinase 9	P14780
MTOR	Serine/threonine-protein kinase mTOR	P42345
NOS2	Nitric oxide synthase, inducible (by homology)	P35228
PIK3CA	PI3-kinase p110-alpha subunit	P42336
PTPN11	Protein-tyrosine phosphatase 2C	Q06124
SERPINA6	Corticosteroid binding globulin	P08185
SHBG	Testis-specific androgen-binding protein	P04278

### 3.4. Protein–protein interactions network results

The top 20 action targets of Resinacein S for NAFLD were put into the STRING database. Moreover, the PPI network was obtained according to the method described in 2.5. Data from the STRING database was put into Cytoscape 3.9.0 for beautification and classification according to the degree value. The degree values were classified into 0–6 and 7–16, and the protein–protein interaction network was generated (Figure 3B).

### 3.5. Resinacein S induced gene expression profiles in human liver cells

**Figure 4:**
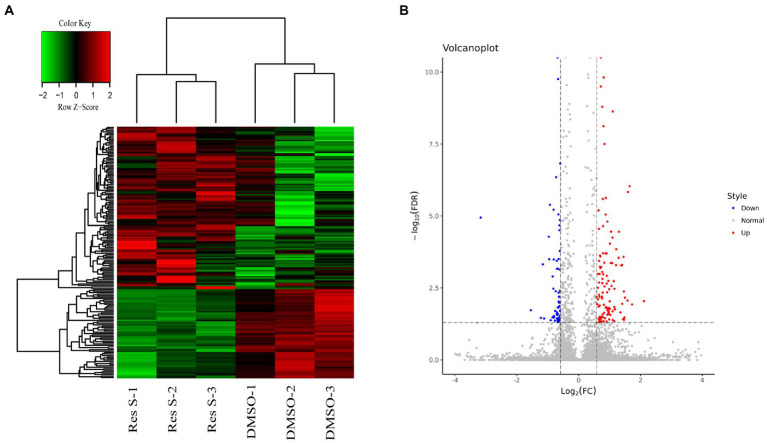
**(A)** The heatmap of the DEGs between Resinacein S treated group and DMSO group. **(B)** The volcano plot of the DEGs between Resinacein S treated group and DMSO group.

In order to further investigate the hub regulated genes of Resinacein S against NAFLD in human liver cells, we treated the human normal liver cell line L02 with Resinacein S and detected the gene expression profiles by RNA-Seq. According to log2FC > 0.585 or < −0.585 and FDR < 0.05, we obtained 172 DEGs (111 up-regulated and 61 down-regulated) in Resinacein S treated group compared to DMSO group (Figure 4, Supplementary Table 1).

### 3.6. Functional enrichment analysis of Resinacein S induced genes in human liver cells

**Figure 5:**
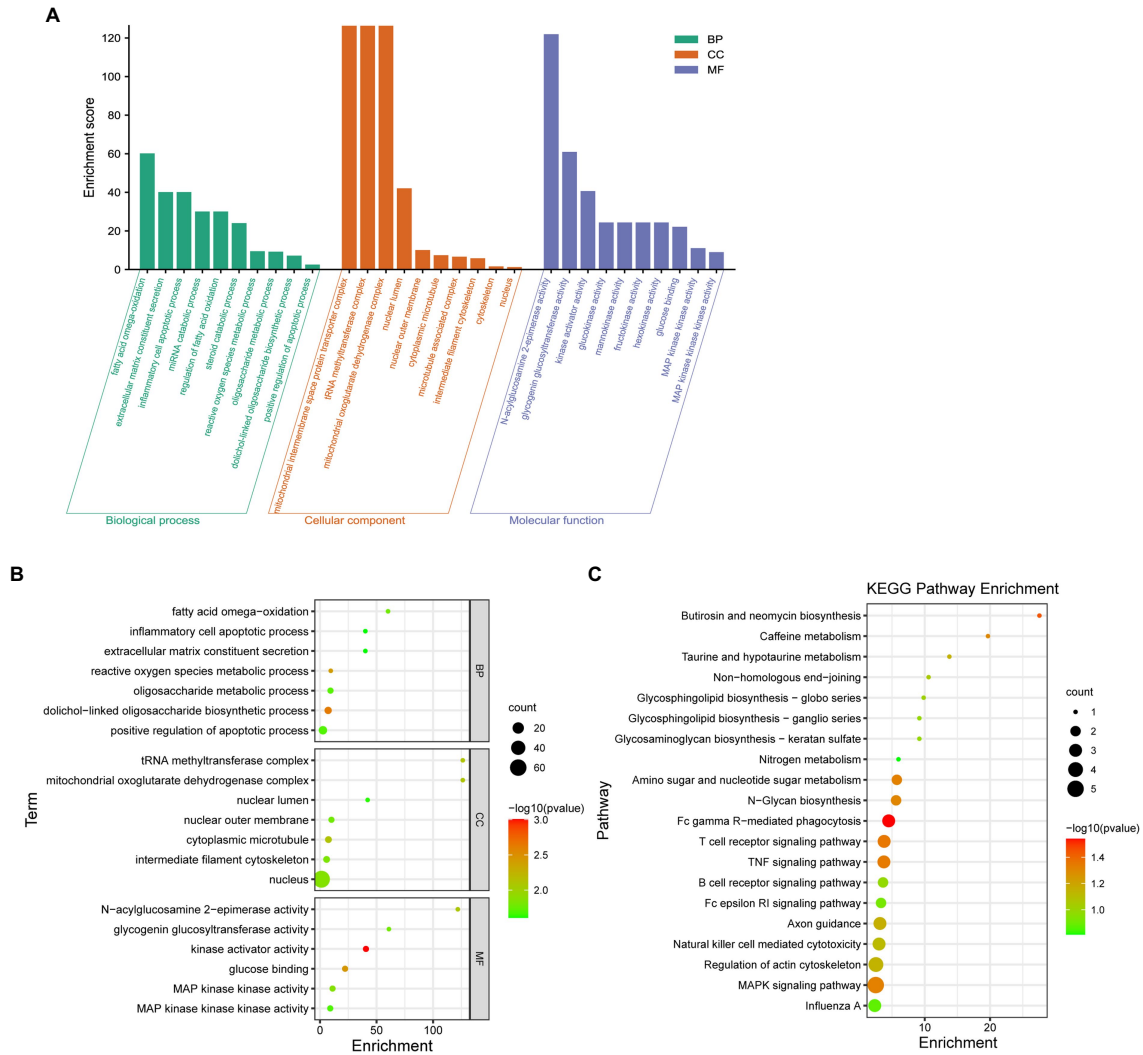
GO and KEGG pathway enrichment analysis was conducted with Resinacein S induced genes in human liver cells. **(A)** The categories in GO terms included cellular components (CC), molecular function (MF), and biological process (BP) were analyzed. **(B)** The top 20 significant GO terms of the overlapped DEGs were chosen based on the order of *p* value from small to large. **(C)** KEGG pathway enrichment analysis was performed with Resinacein S-induced genes. The top 20 significant KEGG pathways of the DEGs were chosen based on the order of *p* value from small to large.

To study the biological function of Resinacein S induced genes in human liver cells, GO annotation was conducted. The significantly enriched GO terms in cellular component (CC), molecular function (MF) and biological process (BP) are shown in Figure 5A and Table 3. Significantly enriched GO terms associated with cellular components included mitochondrial oxoglutarate dehydrogenase complex, tRNA methyltransferase complex, mitochondrial oxoglutarate dehydrogenase complex, nuclear lumen, nuclear outer membrane, cytoplasmic microtubule, microtubule-associated complex, intermediate filament cytoskeleton, cytoskeleton, and nucleus. Significantly enriched GO terms associated with molecular function (MF) included N-acylglucosamine 2-epimerase activity, glycogenin glucosyltransferase activity, kinase activator activity, glucokinase activity, mannokinase activity, fructokinase activity, hexokinase activity, glucose binding, MAP kinase kinase activity, and MAP kinase kinase kinase activity. Significantly enriched GO terms associated with biological process (BP) consisted of fatty acid omega-oxidation, extracellular matrix constituent secretion, inflammatory cell apoptotic process, miRNA catabolic process, regulation of fatty acid oxidation, steroid catabolic process, reactive oxygen species metabolic process, oligosaccharide metabolic process, dolichol-linked oligosaccharide biosynthetic process, and positive regulation of the apoptotic process.

**Table 3:** Top 20 significant GO terms of the overlapped DEGs in livers.

Category	GO ID	Term	List	*p* value
MF	GO:0019209	Kinase activator activity	2	0.000981
BP	GO:0006488	Dolichol-linked oligosaccharide biosynthetic process	4	0.002334
MF	GO:0005536	Glucose binding	2	0.0035
BP	GO:0072593	Reactive oxygen species metabolic process	1	0.00383
CC	GO:0005881	Cytoplasmic microtubule	3	0.007664
CC	GO:0009353	Mitochondrial oxoglutarate dehydrogenase complex	1	0.007916
CC	GO:0043527	tRNA methyltransferase complex	1	0.007916
MF	GO:0050121	N-acylglucosamine 2-epimerase activity	1	0.008203
CC	GO:0005634	Nucleus	64	0.013785
MF	GO:0004708	MAP kinase kinase activity	2	0.013861
CC	GO:0045111	Intermediate filament cytoskeleton	3	0.014839
MF	GO:0008466	Glycogenin glucosyltransferase activity	1	0.016339
BP	GO:0010430	Fatty acid omega-oxidation	1	0.016551
CC	GO:0005640	Nuclear outer membrane	2	0.016571
BP	GO:0009311	Oligosaccharide metabolic process	2	0.019557
BP	GO:0043065	Positive regulation of apoptotic process	7	0.020125
MF	GO:0004709	MAP kinase kinase kinase activity	2	0.020507
CC	GO:0031981	Nuclear lumen	1	0.023562
BP	GO:0070278	Extracellular matrix constituent secretion	1	0.024724
BP	GO:0006925	Inflammatory cell apoptotic process	1	0.024724

The top 20 GO terms associated with Resinacein S induced genes in human liver cells were selected according to the order of *value of p* in ascending order (Figure 5B; Table 3). It was found that the DEGs were mainly enriched in the fatty acid omega-oxidation, dolichol-linked oligosaccharide biosynthetic process, glucose binding, inflammatory cell apoptotic process, glycogenin glucosyltransferase activity, and fatty acid omega-oxidation, etc. These functions were closely related to the physiological regulation of liver metabolism, especially the metabolic homeostasis of lipids and cholesterol.

KEGG enrichment analysis was carried out to explore the biological pathways of the Resinacein S-induced DEGs (Figure 5C; Table 4). We found that the DEGs were significantly enriched in KEGG pathways, including T cell receptor signaling pathway, N-Glycan biosynthesis, Amino sugar and nucleotide sugar metabolism, Glycosphingolipid biosynthesis-globo series, MAPK signaling pathway, and TNF signaling pathway, etc.

**Table 4:** Top 20 significant enriched pathways of the overlapped DEGs in livers.

Category	Pathway ID	*p* value	Genes
Fc gamma R-mediated phagocytosis	PATH:04666	0.028775	VAV3/SCIN/LIMK2
Butirosin and neomycin biosynthesis	PATH:00524	0.035794	HKDC1
T cell receptor signaling pathway	PATH:04660	0.044183	MAP3K8/VAV3/MALT1
TNF signaling pathway	PATH:04668	0.045195	MAP3K8/FAS/MAP2K6
Amino sugar and nucleotide sugar metabolism	PATH:00520	0.047149	RENBP/HKDC1
MAPK signaling pathway	PATH:04010	0.047367	MAP3K8/FAS/MAP2K5 MAP2K6/CACNA1H
N-Glycan biosynthesis	PATH:00510	0.048932	ALG9/DOLPP1
Caffeine metabolism	PATH:00232	0.049758	NAT1
Axon guidance	PATH:04360	0.069072	LIMK2/ABLIM3/NTN4
Taurine and hypotaurine metabolism	PATH:00430	0.070332	CSAD
Regulation of actin cytoskeleton	PATH:04810	0.071639	VAV3/SCIN/LIMK2 IQGAP2
Natural killer cell mediated cytotoxicity	PATH:04650	0.076769	FAS/VAV3/ULBP3
Non-homologous end-joining	PATH:03450	0.09047	POLM
Glycosphingolipid biosynthesis - globo series	PATH:00603	0.097087	ST3GAL1
Glycosaminoglycan biosynthesis - keratan sulfate	PATH:00533	0.103657	ST3GAL1
Glycosphingolipid biosynthesis - ganglio series	PATH:00604	0.103657	ST3GAL1
B cell receptor signaling pathway	PATH:04662	0.104971	VAV3/MALT1
Fc epsilon RI signaling pathway	PATH:04664	0.121474	VAV3/MAP2K6
Influenza A	PATH:05164	0.133377	FAS/RAE1/MAP2K6
Nitrogen metabolism	PATH:00910	0.154559	CA5B

Overall, these metabolic pathways are closely related to liver metabolic balance, which substantiates the protective effect of Resinacein S against NAFLD.

### 3.7. The hub proteins of Resinacein S regulated against nonalcoholic fatty liver disease

**Figure 6:**
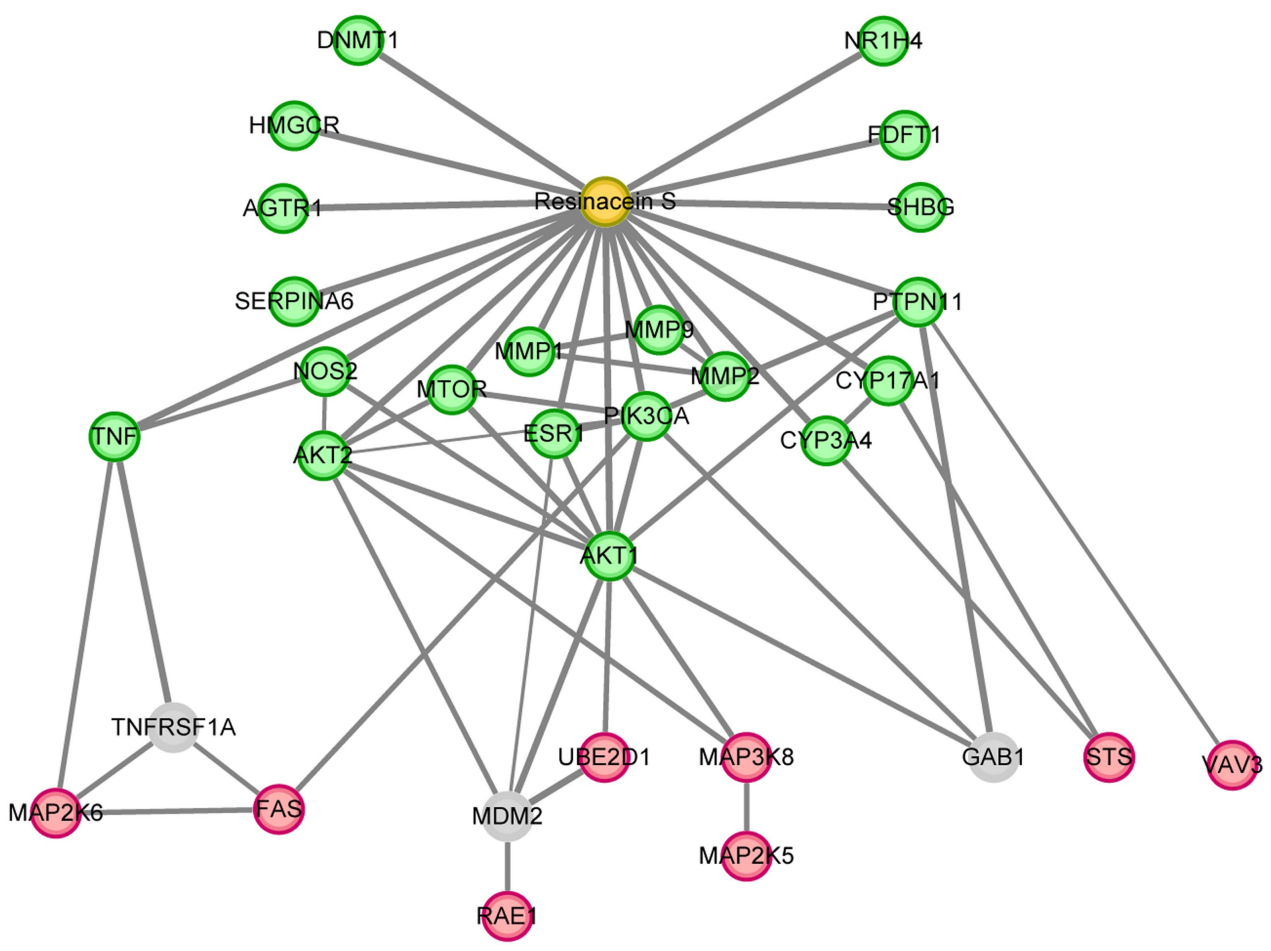
The hub proteins of Resinacein S regulated against NAFLD. The interaction relationship analysis was performed with the DEGs of Resinacein S regulated in human liver cells and the target genes of Resinacein S against NAFLD from public database. DEGs of Resinacein S regulated in human normal liver cells were shown in red circle, and the 20 target genes of Resinacein S against NAFLD from public database were shown in green. The analysis was generated by Cytoscape 3.9.0.

To determine the hub proteins that mediate the protective role of Resinacein S against NAFLD in human liver cells, we analyzed the interaction relationships of 172 DEGs of Resinacein S regulated in human normal liver cell line L02 according to RNA-Seq (Figure 3), and 20 target genes of Resinacein S against NAFLD from public database (Figure 4). The relationships were obtained by the STRING database, including evidence from experiments, databases, and co-expression data, and binding scores greater than 0.7 (high confidence). Therefore, according to the STRING analysis, the Resinacein S-mediated junction proteins against NAFLD were TNF, PIK3CA, AKT1, AKT2, ESR1, CYP3A4, CYP17A1, and PTPN11 (Figure 6). Among them, AKT1 and AKT2 play an important role in the interaction relationships which indicated the regulation of AKT pathway by Resinacein S against NAFLD.

## 4. Discussion

NAFLD is widely thought to be the most common chronic liver disease, concomitant with the global obesity epidemic. During the development and progress of NAFLD, liver steatosis is considered a benign state, while NASH exhibits chronic progressive liver damage. Fatty degeneration is related to inflammation and fibrosis and gradually develops into cirrhosis. The pathogenesis of NAFLD is closely related to dysregulated metabolism in obese patients, excess free fatty acid (FFA) accumulation and the excessive secretion of several cellular inflammatory mediators (14). There is an increasing consensus that NAFLD is a heterogeneous disease associated with multiple-hit pathogenesis yielding different phenotypes. Although the clinical presentation of NAFLD can be heterogeneous, insulin resistance plays a leading role in NAFLD (15).

In this study, we focused on the role of the Resinacein S compound extracted from *G. resinaceum* in treating NAFLD patients. First, we identified the extracted triterpenoid compounds and obtained the molecular structure of Resinacein S through mass spectrometry analysis. By interaction relationship analysis of the DEGs of Resinacein S regulated in human liver cells and the target genes of Resinacein S against NAFLD from public database, we found 8 proteins TNF, PIK3CA, AKT1, AKT2, ESR1, CYP3A4, CYP17A1, and PTPN11 may be the hub proteins of Resinacein S regulated against NAFLD.

According to former studies, Resinacein S treatment can dramatically induce the expression of thermogenesis related genes such as Ucp1 and Pgc1α, fatty acid oxidation related genes such as Pparα and Cpt1α, lipolysis related genes such as Hsl and Atgl, and mitochondriogenesis-related genes. Resinacein S may be involved in activation of AMPK/PGC1α signaling pathway (6). Now we obtained eight Resinacein S targeting genes with potential therapeutic effects against NAFLD through RNA seq analysis and PPI network analysis.

Obesity is related to the increase of circulating Tumor Necrosis Factor (TNF, TNF-α), a pro-inflammatory cytokine that induces the death of liver cells (16). In the process of inflammation, TNF is one of the main pro-inflammatory cytokines, which regulates innate immunity and adaptive immune response (17). Clinical evidence shows that the level of circulating TNF-α in patients with nonalcoholic steatohepatitis (NASH) is highly correlated with the degree of liver fibrosis (18). AKT Serine/Threonine Kinase 1 (AKT1) is a serine/threonine protein kinase, which is called an important downstream target signal pathway of insulin and has anti-apoptosis and peripheral metabolic effects. Studies have shown that the inhibition of ROS production mediated by AKT1 inhibits the fibrosis transformation from NAFLD to liver (19). AKT1 deficiency led to the inhibition of AKT/mTOR/S6K signaling pathway in hepatocytes, which was crucial for the development of hepatic steatosis and provided a new scheme for the development of NAFLD therapy (20). Studies have shown that AKT Serine/Threonine Kinase 2 (AKT2) is essential for lipid synthesis in the liver (21). It was found that hepatocyte-specific Phosphatase and Tensin Homolog (PTEN) deficiency in mice showed liver steatosis due to over-activation of AKT2 (22). As the precursor of NAFLD, deleting AKT2, the downstream target of PTEN signal, can block the development of NASH and reduce the development of liver fibrosis, which also reduces the occurrence of NAFLD from another development process (23). Phosphatidylinositol-4,5-Bisphosphate 3-Kinase Catalytic Subunit Alpha (PIK3CA) is an important component of PI3K/AKT/mTOR pathway. PIK3CA mutated cells showed inflated induction of the *de novo* lipogenesis transcriptional regulator SREBP1 and elevated exogenous FA uptake capacity both of which can lead to lipid-enriched phenotype (24, 25). ESR1 is a nuclear and membrane hormone receptor. Previous studies showed that ESR1 could negatively regulate hepatocyte pyroptosis by directly interacting with gasdermin D (GSDMD). ESR1 deficiency could induce pyroptosis, impaired glucose tolerance, and reduce lipid accumulation in hepatocytes (26–28). Cytochrome P450 Family 3 Subfamily A Member 4 (CYP3A4) is involved in the metabolism of sterols, retinoids and fatty acids. The activity of CYP3A4 is usually reduced in mouse and cell models of NAFLD, also in human. In NAFLD-model mice, the luciferase activity of CYP3A4 from livers was about 38% lower than the normal ones (29, 30). Cytochrome P450 Family 17 Subfamily A Member 1 (CYP17A1) is an important enzyme for Dehydroepiandrosterone (DHEA) synthesis. Former evidences showed that DHEA can modulate oxidative stress, insulin resistance, and fibrosis observed in serious NAFLD (31, 32). Protein Tyrosine Phosphatase Non-Receptor Type 11 (PTPN11) is the first identified oncogenic tyrosine phosphatase which can cooperate with PTEN to maintain the liver homeostasis and function. PTPN11 in hepatocytes could induce early-onset non-alcoholic steatohepatitis (NASH) (33).

Besides, according to our KEGG enrichment analysis, we revealed that the targets of Resinacein S mainly focused on T cell receptor signaling pathway, N-Glycan biosynthesis, Amino sugar and nucleotide sugar metabolism, Glycosphingolipid biosynthesis-globo series, MAPK signaling pathway, and TNF signaling pathway.

In conclusion, we have revealed the structure of Resinacein S and demonstrated that Resinacein S could significantly attenuate high-fat diet induced hepatic steatosis and hepatic lipid accumulation. We have also developed a new gene expression feature related to NAFLD by using Resinacein S-dependent DEGs, especially hub proteins in PPI network analysis, which can assist in diagnosing and treating NAFLD populations as well as drug discovery and development in the near future. Nevertheless, although the extracts of *Ganoderma* are usually safe and most of the side effects are very mild, the side effect of Resinacein S deserves further studies especially on human. Besides, the molecular mechanisms of Resinacein S against NAFLD and *in vivo* models for further proving the metabolic phenotypes of Resinacein S need to be further investigated.

## 5. Conclusion

Overall, Resinacein S yields a protective effect against steatosis and liver injury and can significantly change the gene expression profile in fatty liver cells. Functional enrichment analysis showed significant enrichment in lipid and glucose metabolism. In addition, intersected genes between NAFLD and Resinacein S-induced DEGs, especially the hub protein in PPI network analysis, can be used to characterize NAFLD-related gene expression characteristics for NAFLD diagnosis, treatment and drug development.

## Data availability statement

The data presented in the study are deposited in the GEO repository, accession number GSE223990.

## Ethics statement

The animal study was reviewed and approved by Animal Care and Use Committee of Shanghai Tenth People’s Hospital of Tongji University.

## Author contributions

S-QC, GW, and F-FM conceived the project. The data analysis was done by FM, S-SG, and Y-JH. F-FM and S-SG wrote the drafts of the manuscript. Y-JH checked and revised the manuscript. NZ and J-KF participated in collection of the published datasets. Z-HL, Y-QZ, and L-YY gave many suggestions about the manuscript writing. All authors contributed to the article and approved the submitted version.

## Funding

This work was partially supported by grants from the National Natural Science Foundation of China (Nos. 82002480, 8200032).

## Conflict of interest

The authors declare that the research was conducted in the absence of any commercial or financial relationships that could be construed as a potential conflict of interest.

## Publisher’s note

All claims expressed in this article are solely those of the authors and do not necessarily represent those of their affiliated organizations, or those of the publisher, the editors and the reviewers. Any product that may be evaluated in this article, or claim that may be made by its manufacturer, is not guaranteed or endorsed by the publisher.

## Footnotes

^1^ http://disgenet.org/home/

^2^ https://www.genecards.org/

^3^ https://omim.org/

^4^ https://bioinfogp.cnb.csic.es/tools/venny/index.html

^5^ https://pubchem.ncbi.nlm.nih.gov

^6^ https://swisstargetprediction.ch/

^7^ https://www.uniprot.org

^8^ https://string-db.org/
