# Supplementary material for: Network pharmacology-based analysis of Resinacein S against non-alcoholic fatty liver disease by modulating lipid metabolism
Source: Front Nutr. 2023 Feb 14;10:1076569. doi: 10.3389/fnut.2023.1076569 (PMC9971728; doi:10.3389/fnut.2023.1076569)
Supplement: Supplementary file 1 [file Data_Sheet_1.zip › Supplemental data/Supplemental data.docx]

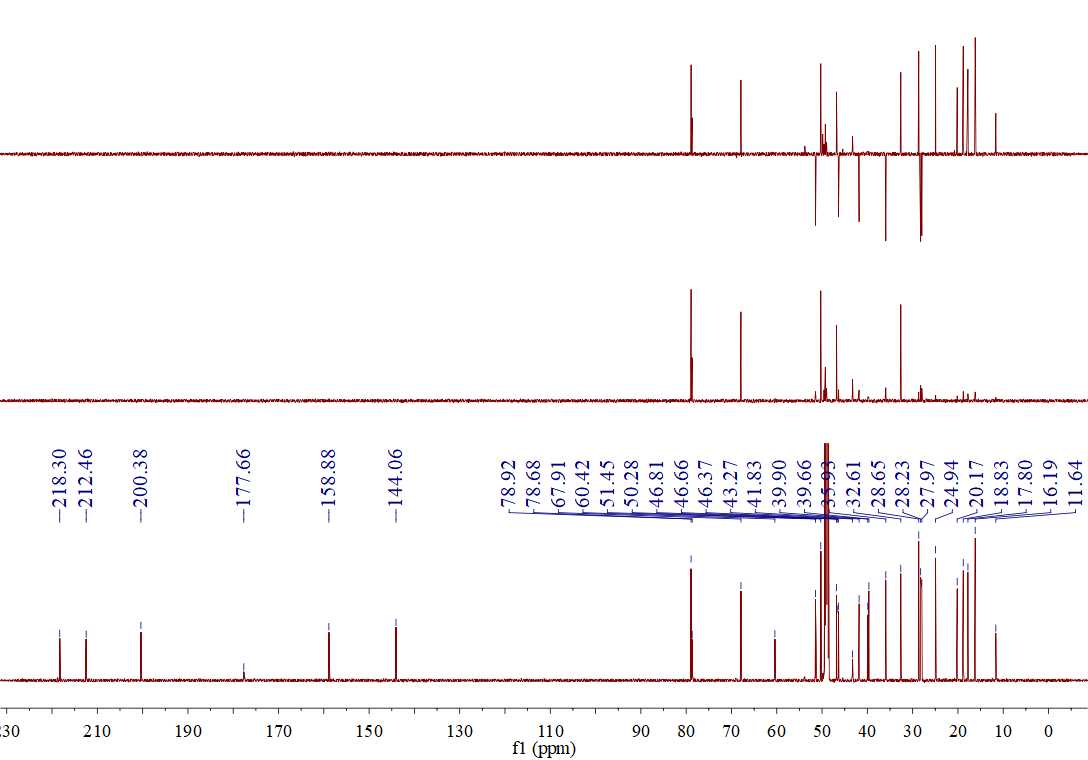


**Supplemental Figure 1.** ^13^C NMR and DEPT spectra (150 MHz) of Resinacenin S in CD_3_OD.


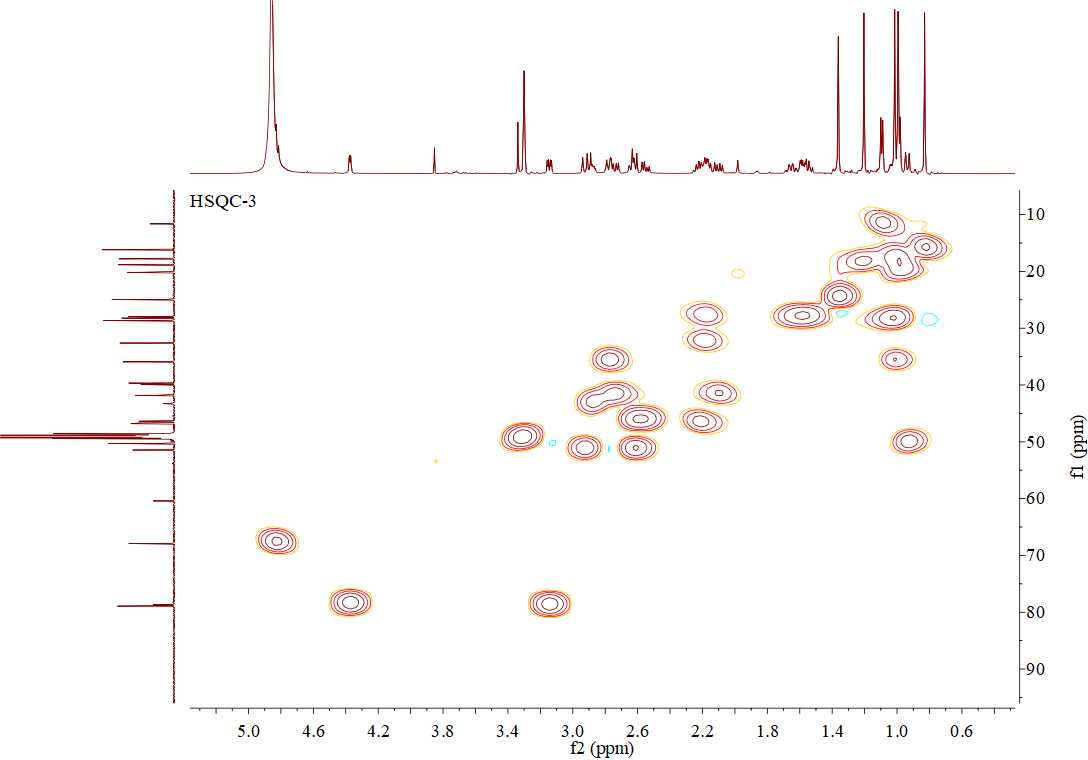


**Supplemental Figure 2.** HSQC spectrum of Resinacenin S in CD_3_OD.


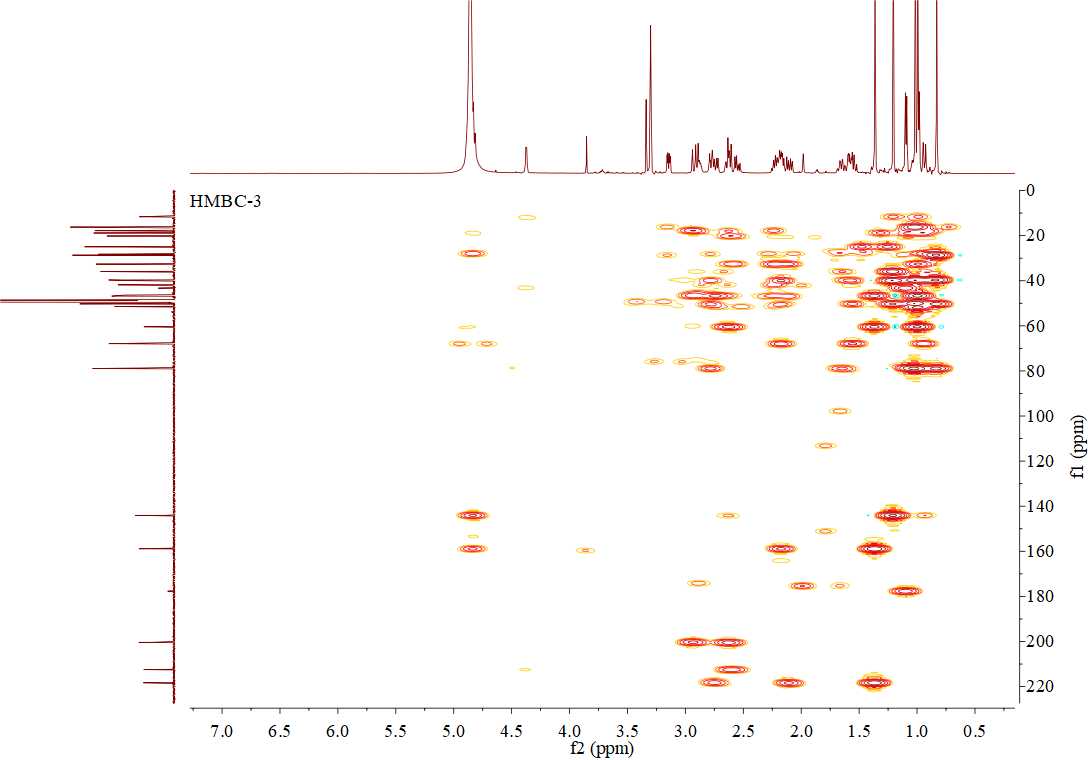


**Supplemental Figure 3.** HMBC spectrum of Resinacenin S in CD_3_OD.


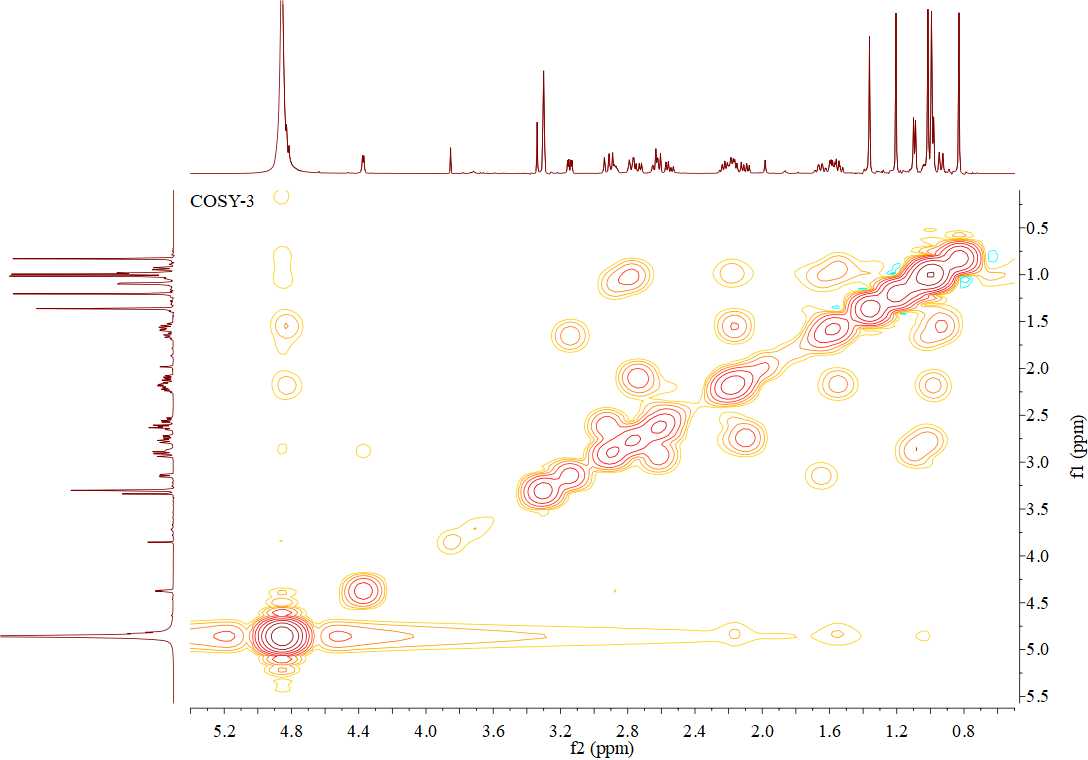


**Supplemental Figure 4.** ^1^H-^1^H COSY spectrum (600 MHz) of Resinacenin S in CD_3_OD.


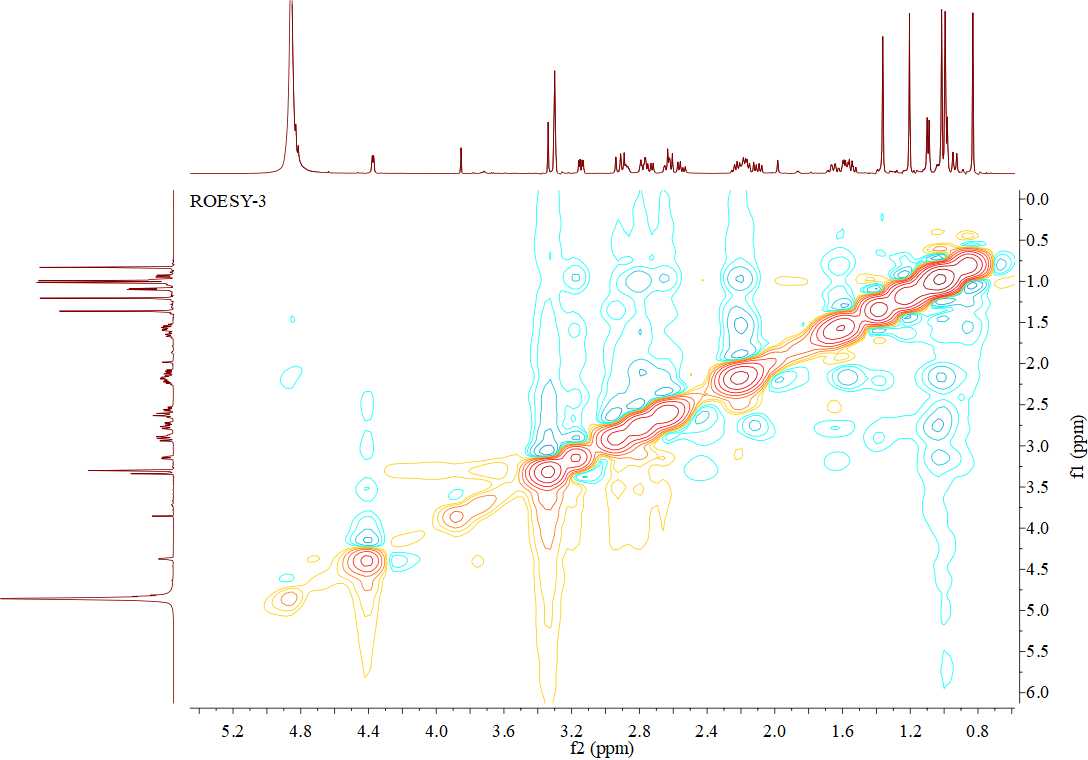


**Supplemental Figure 5.** ROESY spectrum (600 MHz) of Resinacenin S in CD_3_OD.


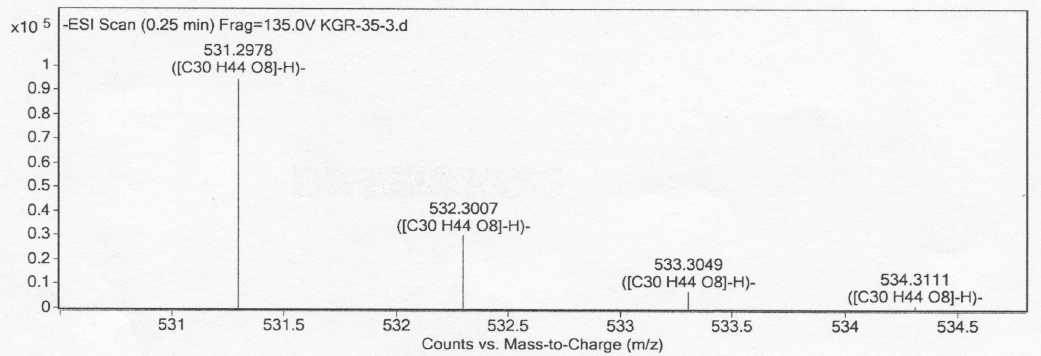


**Supplemental Figure 6.** HRESIMS spectrum of Resinacenin S.
