# Supplementary figures and images for: Network pharmacology-based analysis of Resinacein S against non-alcoholic fatty liver disease by modulating lipid metabolism
Source: Front Nutr. 2023 Feb 14;10:1076569. doi: 10.3389/fnut.2023.1076569 (PMC9971728; doi:10.3389/fnut.2023.1076569)

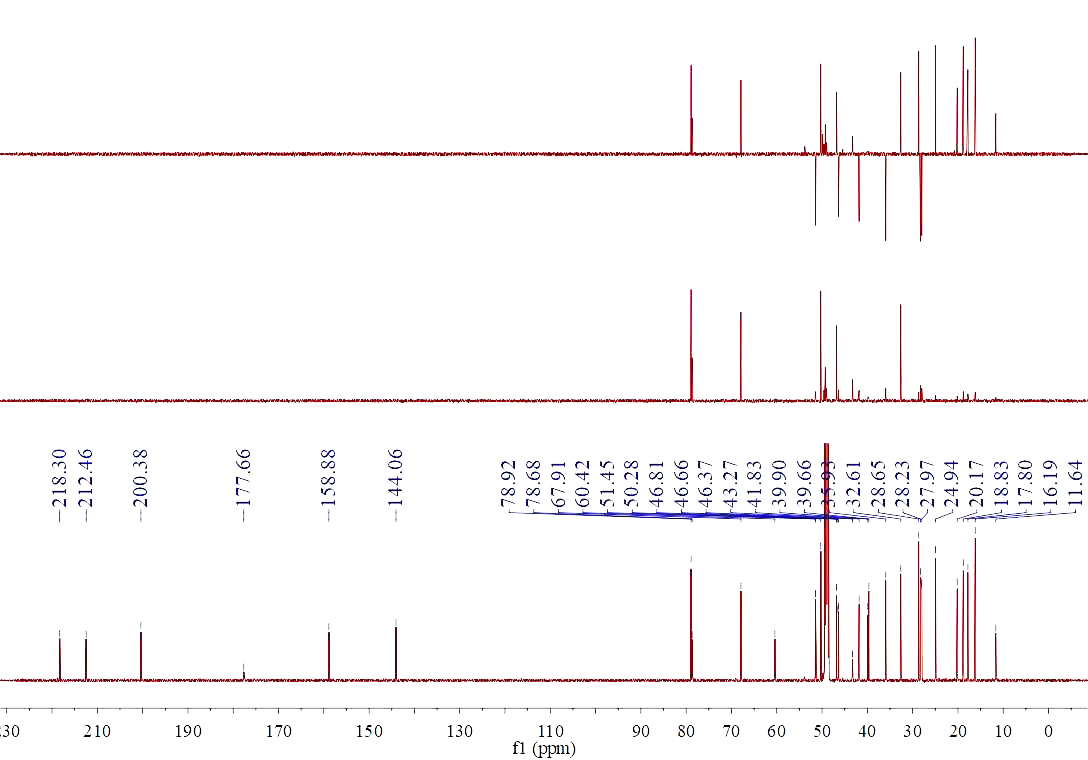

Supplement: Supplementary file 1 [file Data_Sheet_1.zip › Supplemental data/Supplemental Figure 1.tif]

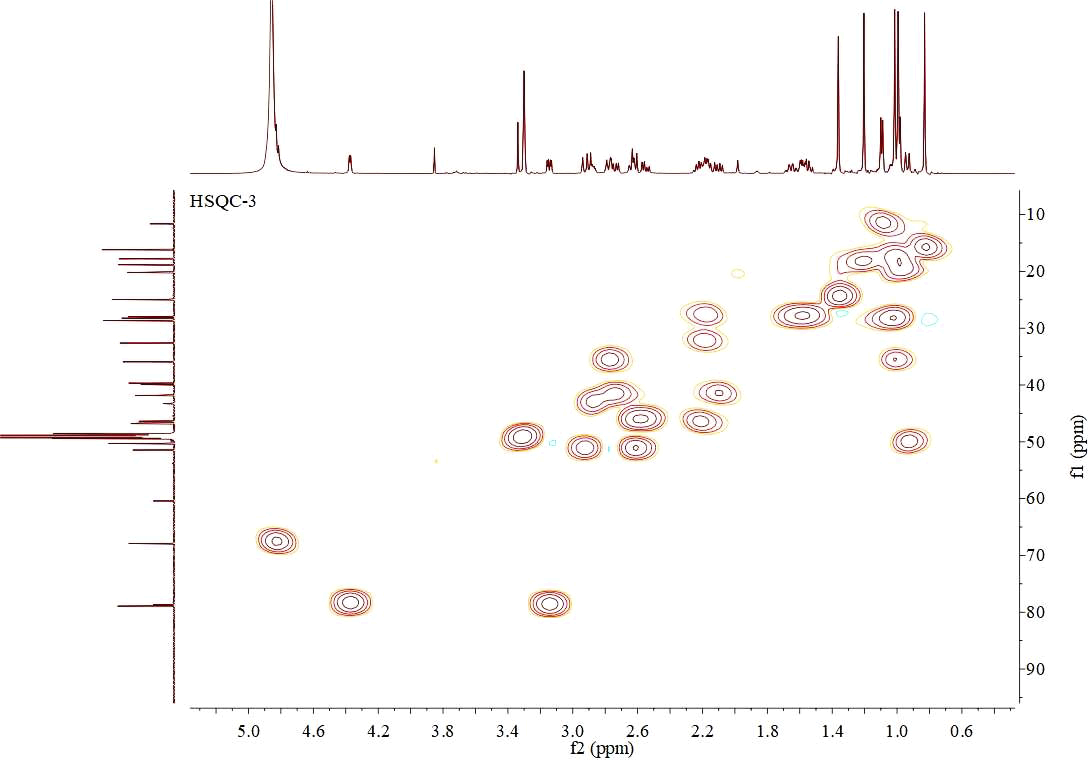

Supplement: Supplementary file 1 [file Data_Sheet_1.zip › Supplemental data/Supplemental Figure 2.tif]

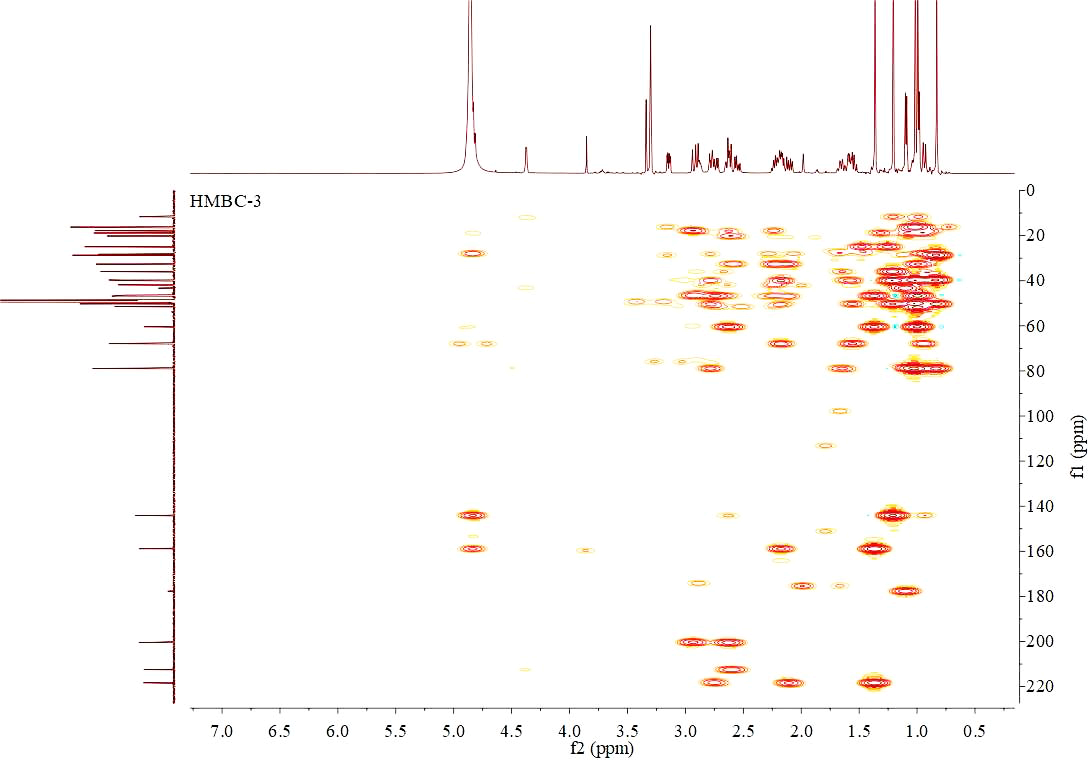

Supplement: Supplementary file 1 [file Data_Sheet_1.zip › Supplemental data/Supplemental Figure 3.tif]

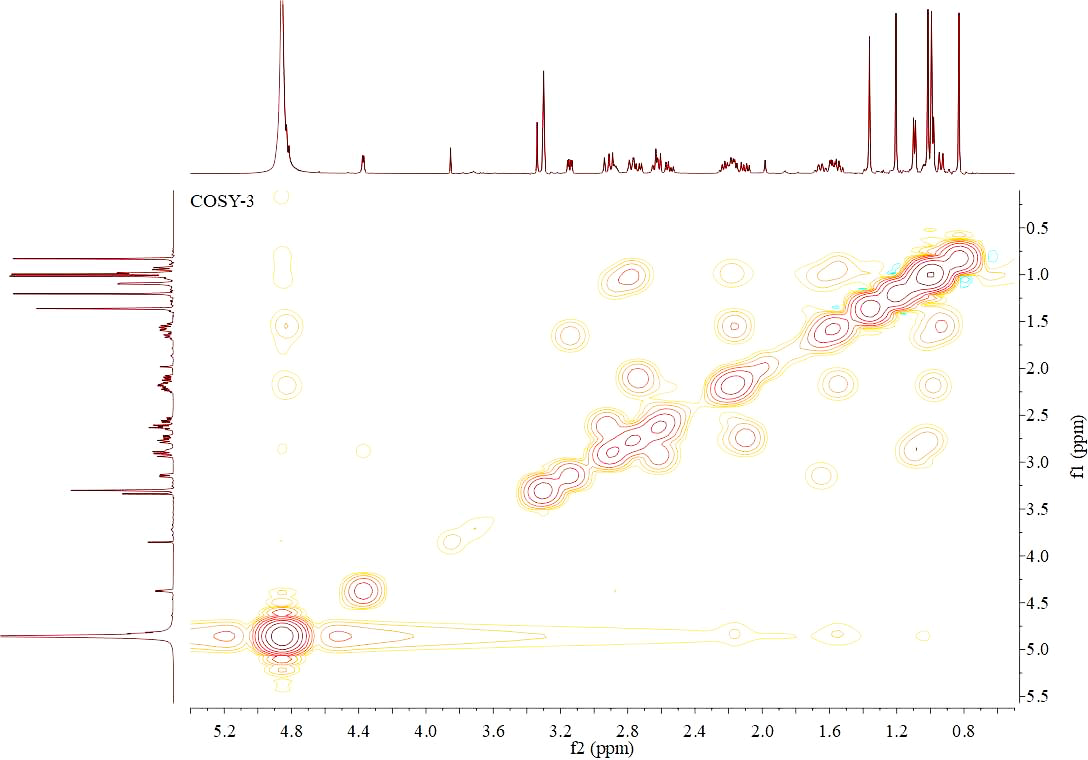

Supplement: Supplementary file 1 [file Data_Sheet_1.zip › Supplemental data/Supplemental Figure 4.tif]

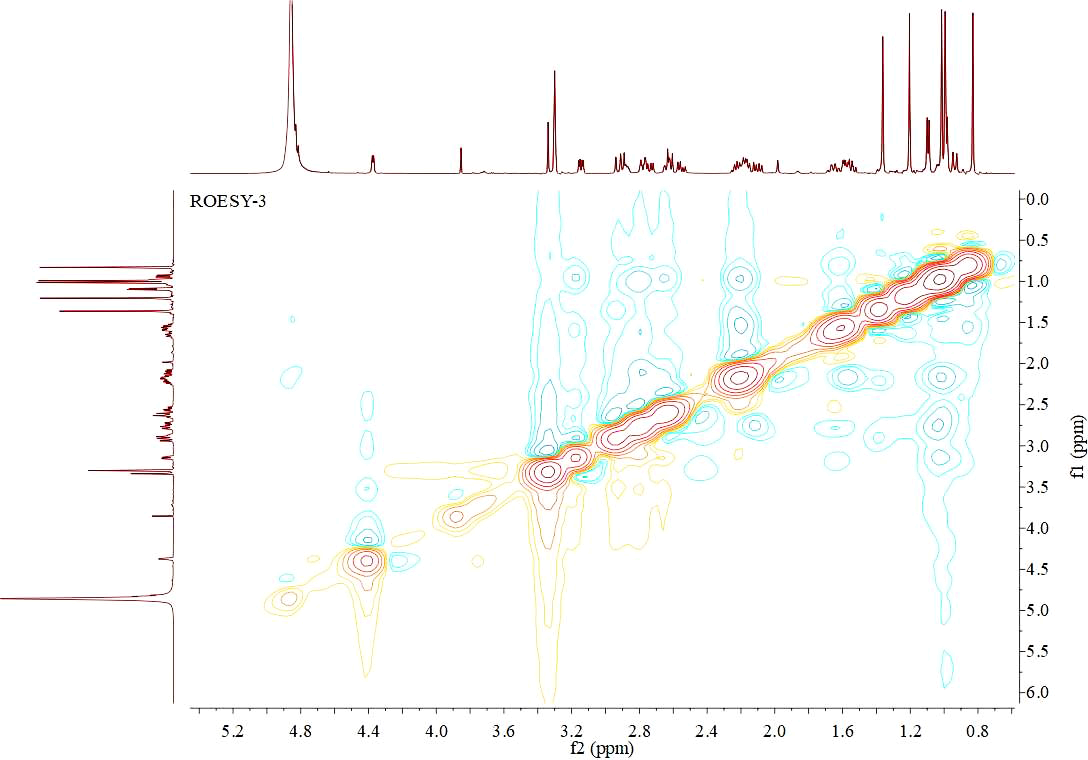

Supplement: Supplementary file 1 [file Data_Sheet_1.zip › Supplemental data/Supplemental Figure 5.tif]

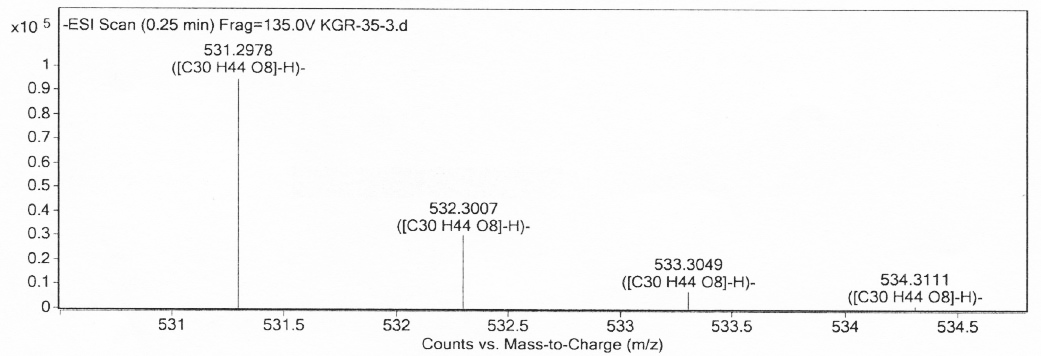

Supplement: Supplementary file 1 [file Data_Sheet_1.zip › Supplemental data/Supplemental Figure 6.tif]
